# Supplementary material for: Attitudes and Opinions of Young Gynecologists on Pregnancy Termination: Results of a Cross-Sectional Survey in Poland
Source: Int J Environ Res Public Health. 2020 May 31;17(11):3895. doi: 10.3390/ijerph17113895 (PMC7311986; doi:10.3390/ijerph17113895)
Supplement: Supplementary file 1 [file ijerph-17-03895-s001.pdf]

1st Department of Obstetrics and Gynecology
ŻELAZNA MEDICAL CENTER
St Sophia's Specialist Hospital in Warsaw
ul. Żelazna 90, 01-004 Warszawa
tel. 22 25 59 801, e-mail: szpital@szpitalzelazna.pl
Head of Department: Prof. Grzegorz Jakiel, MD, PhD

Dear Sir/Madam, we kindly ask you to complete this anonymous questionnaire.

Data included in the questionnaire will be used in scientific research and the evaluation of medical services provided by our facility.

Thank you for taking the time to complete this questionnaire.

GENERAL INFORMATION

1. Physician's age:

………………………………………………………………………………. *(Insert the number of completed years of life)*

2. Physician's sex:

a woman b man

3. Place of residence:

a province capital b district capital c other towns d village

4. Which province do you live in? ………………………………………………………………….

5. Workplace:

a teaching hospital b provincial hospital c district hospital d other hospitals

6. Education

a MD b MD PhD c MD PhD (post-doctoral) d Professor

7. Marital status:

a unmarried b married c divorced d widowed

8. Do you have children?

a Yes b No

9. Job seniority in the profession of a gynecologist-obstetrician:

………………………………………………………………………………………………………. *(Please insert the number of years)*

II. RELIGION

1. What is your denomination?

a Catholicism

b Protestantism

c Islam

d Judaism

e No religious belief

f Others………………………………………………………………………………………. *(Specify your denomination)*

2. How would you describe your belief?

a practicing b non-practicing c doubting d no religious belief

3. In case of being a believer: How often do you go to church?

a I attend at least one mass a week.

b I attend at least one mass a month.

c I attend a mass several times a year.

d I don't go to church.

III. WORLDVIEW

1. Do you think that the Polish society is intolerant as regards pregnancy termination?

a I strongly agree b I agree c No opinion d I disagree e I strongly disagree

2. Do you prescribe emergency postcoital contraception?

a Yes b No

3. Do you think that *in vitro* fertilization should be covered by the state?

a I strongly agree b I agree c No opinion d I disagree e I strongly disagree

4. What are your political inclinations?

a the left wing b radical right wing c liberal right wing d rural community members

e Others…………………………………………………………………………..

5. Do you perform pregnancy termination procedures?

a Yes b No

6. If you do not perform pregnancy termination procedures, please specify the reason(s). *(Multiple answers possible)*

a It's contrary to my worldview

b No such procedures are performed at my hospital

c I am worried about the opinion of the society

d Others………………………………………………………………………………………

7. Are you for abortion without medical indications?
a Yes, always, until the end of pregnancy
b Yes, until 12 gestational weeks
c Yes, until 22 gestational weeks
d No

8. Are you for abortion in case of severe fetal defects?
a Yes, always, until the end of pregnancy
b Yes, until 12 gestational weeks
c Yes, until 22 gestational weeks
d No

9. Are you for abortion in case of lethal fetal defects?
a Yes, always, until the end of pregnancy
b Yes, until 12 gestational weeks
c Yes, until 22 gestational weeks
d No

10. Are you for abortion in case of a rape?
a Yes, always, until the end of pregnancy
b Yes, until 12 gestational weeks
c Yes, until 22 gestational weeks
d No

11. Are you for abortion in case of a severe life-threatening maternal disease (e.g. cancer)?
a Yes, always, until the end of pregnancy
b Yes, until 12 gestational weeks
c Yes, until 22 gestational weeks
d No

12. Are you for abortion in case of the diagnosis of Down syndrome in the fetus?
a Yes, always, until the end of pregnancy
b Yes, until 12 gestational weeks
c Yes, until 22 gestational weeks
d No

13. In what situation would you personally perform a pregnancy termination procedure? *(Multiple answers possible)*
a In case of a severe fetal defect
b In case of a lethal fetal defect
c In case of a severe maternal disease
d In case of a pregnancy which resulted from a rape
e In case of an unwanted pregnancy (abortion on request)
f Never

14. Do you perform fetal ultrasound?

a Yes b No

15. Do you perform prenatal tests?

a Yes b No

16. If you do not perform pregnancy terminations personally – if you diagnose defects, do you refer the patient to a facility where such procedures are performed?

a Yes b No

17. If you do not refer patients for pregnancy termination procedures, please specify the reason(s): *(Multiple answers possible)*

a I don't know such centers

b Referring a patient for a termination is contrary to my conscience

c I am worried about the opinion of the society

d Others……………………………………………………………………………………….

18. Would you opt for a pregnancy termination if a lethal defect was diagnosed in your child?

a Definitely yes b Yes c No opinion d No e Definitely no

19. Would you opt for a pregnancy termination in case of your own child if a life-threatening maternal condition was diagnosed?

a Definitely yes b Yes c No opinion d No e Definitely no

20. Do you think that performing a Caesarean section due to fetal indications (e.g. no progress in delivery, occiput posterior position) in case of a lethal fetal defect is inappropriate management?

a Definitely yes b Yes c No opinion d No e Definitely no

IV. FINAL REMARKS

Comments……
……
……
…………………………………………………………………………………

Thank you for completing the questionnaire.
